# A seroepidemiologic study of Reston ebolavirus in swine in the Philippines

**DOI:** 10.1186/1746-6148-8-82

**Published:** 2012-06-18

**Authors:** Yusuke Sayama, Catalino Demetria, Mariko Saito, Rachel R Azul, Satoshi Taniguchi, Shuetsu Fukushi, Tomoki Yoshikawa, Itoe Iizuka, Tetsuya Mizutani, Ichiro Kurane, Fidelino F Malbas, Socorro Lupisan, Davinio P Catbagan, Samuel B Animas, Rieldrin G Morales, Emelinda L Lopez, Karen Rose C Dazo, Magdalena S Cruz, Remigio Olveda, Masayuki Saijo, Hitoshi Oshitani, Shigeru Morikawa

**Affiliations:** 1 Department of Virology 1, National Institute of Infectious Diseases, 4-7-1 Gakuen, Musashimurayama, Tokyo, 208-0011, Japan; 2 Department of Virology, Tohoku University Graduate School of Medicine, 2-1 Seiryo-machi, Aoba-ku, Sendai, Miyagi, 980-8575, Japan; 3 Tohoku-RITM Collaborating Research Center for Emerging and Reemerging Infectious Diseases, FILINVEST Corporate City, Alabang, Muntinlupa City, 1781, Philippines; 4 Research Institute for Tropical Medicine, FILINVEST Corporate City, Alabang, Muntinlupa City, 1781, Philippines; 5 Bureau of Animal Industry, Elliptical Road, Diliman, Quezon City, 1107, Philippines

**Keywords:** Reston ebolavirus, Antibody, Swine, Philippines

## Abstract

**Background:**

Ebola viruses cause viral hemorrhagic fever in humans and non-human primates and are endemic in Africa. Reston ebolavirus (REBOV) has caused several epizootics in cynomolgus monkeys (*Macaca fascicularis*) but is not associated with any human disease. In late 2008, REBOV infections were identified in swine for the first time in the Philippines.

**Methods:**

A total of 215 swine sera collected at two REBOV-affected farms in 2008, in Pangasinan and Bulacan, were tested for the presence of REBOV-specific antibodies using multiple serodiagnosis systems. A total of 98 swine sera collected in a non-epizootic region, Tarlac, were also tested to clarify the prevalence of REBOV infection in the general swine population in the Philippines.

**Results:**

Some 70 % of swine sera at the affected farms were positive for REBOV antibodies in the multiple serodiagnosis systems. On the other hand, none of the swine sera collected in Tarlac showed positive reactions in any of the diagnosis systems.

**Conclusions:**

The high prevalence of REBOV infection in swine in the affected farms in 2008 suggests that swine is susceptible for REBOV infection. The multiple serological assays used in the study are thought to be useful for future surveillance of REOBV infection in swine in the Philippines.

## Background

The Ebola virus (EBOV) is an enveloped, negative-strand RNA virus belonging to the family *filoviridae* in the order of *mononegavirale*[1]. Four of the five ebolavirus species, Zaire (ZEBOV), Sudan, Tai Forest, and the recently discovered Bundibugyo ebolavirus, are endemic in continental Africa and cause a severe form of viral hemorrhagic fever with high mortality in humans and non-human primates [2-6]. Reston ebolavirus (REBOV) is sporadic in the Philippines and has caused several epizootics in cynomolgus macaques [7]. REBOV was first isolated in 1989 from cynomolgus macaques imported from the Philippines for medical research in the United States [7-10]. About 1,000 monkeys died or were euthanized in a quarantine facility in Reston, Virginia. Subsequently, 21 animal handlers at the Philippine exporter and four employees of the quarantine facility were found to have antibodies to the virus, indicating that they had been infected [11,12]. Epizootics in monkeys in the Philippines were then reported in 1992 and 1996, and all the epizootics have been traced back to a single monkey facility, in Calamba, Laguna in the Philippines [11,13-16]. Since the closure of the facility in 1997, no REBOV epizootics in cynomolgus monkeys have been reported.

In October 2008, REBOV infection was confirmed for the first time in swine associated with multiple epizootics of respiratory and abortion-related diseases in the Philippines [17]. In several pools of swine samples collected from geographically distant swine farms, co-infection with REBOV and porcine reproductive and respiratory syndrome virus (PRRSV) was confirmed [17]. Serological studies of limited scale on 13 swine sera in the affected farms failed to detect REBOV antibodies in ELISA, although PRRSV antibodies were detected [17]. It is still unclear how REBOV was spread among swine during the epizootic. Moreover, it is not clear if REBOV infection in the swine population is either sporadic and incidental or common in the Philippines. To try to answer these questions, we prepared multiple serodiagnosis systems for detecting REBOV infection in swine and analyzed swine sera obtained from the affected farms and from farms not associated with any epizootics in the Philippines. The results showed a high prevalence of REBOV infection in swine in the affected farms at the epizootics in 2008; however, REBOV antibodies were not detected in the swine population not associated with the epizootics, indicating that REBOV infection in swine in the Philippines is not common, at least in some parts of Tarlac.

## Results

### Detection of REBOV-NP and -GP antibodies in swine using IFA

**Figure 1:**
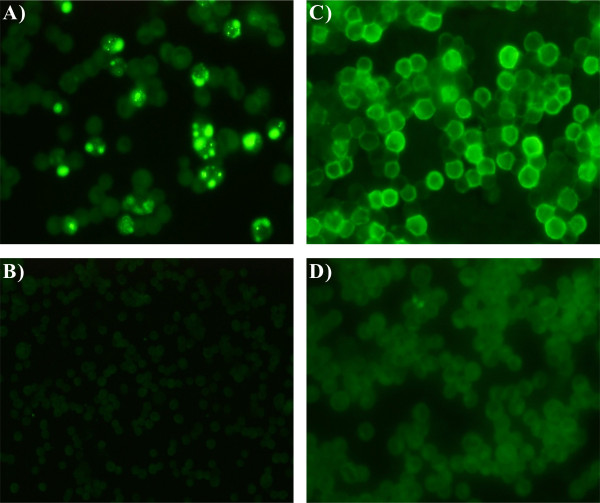
**Detection of REBOV antibodies in IFA.** Immunofluorescence staining pattern of HeLa cells expressing REBOV-NP (**A**) and REBOV-GP (**C**) with a REBOV antibody positive swine serum at a dilution of 1 in 160. Negative staining pattern of HeLa cells expressing REBOV-NP (**B**) and REBOV-GP (**D**) with a REBOV antibody negative swine serum (B and D).

Swine sera were analyzed for the presence of REBOV-NP and -GP antibodies in IFA specific to REBOV-NP and -GP, respectively. In the IFA, none of the 49 swine sera collected in Japan showed a positive reaction (data not shown), and so they were considered to be REBOV-NP and -GP antibody negative. In the IFA specific to REBOV-NP, antibody positive swine sera showed characteristic granular staining patterns in the cytoplasm (Figure 1A), which were indistinguishable from those of REBOV-infected cynomolgus monkey sera [18] and REBOV-NP immunized rabbit sera (data not shown). Antibody-negative swine sera showed no reaction (Figure 1B). In the IFA specific to REBOV-GP, antibody positive swine sera showed characteristic cellular surface staining patterns (Figure 1C), which were indistinguishable from those of REBOV-infected cynomolgus monkey sera and REBOV-GP immunized rabbit sera (data not shown). Antibody-negative swine sera showed no reaction (Figure 1D). In Bulacan, 104 (71.2%) and 115 (78.8%) of the 146 swine sera showed positive reactions in the NP- and GP-specific IFA, respectively. In Pangasinan, each 54 (78.3%) of the 69 swine sera showed positive reactions in the NP- and GP-specific IFA. In total, 158 (73.5%) and 169 (78.6%) of the 215 swine sera collected at the affected farms were REBOV-NP and -GP antibody positive in the IFA, respectively (Table 1). On the other hand, none of the 98 swine sera collected in Tarlac in the Philippines showed any positive reaction (Table 1).

**Table 1:** Detection of REBOV-antibodies in swine

	**NP**		**GP**		**NT**
	**IgG-ELISA**	**IFA**	**IgG-ELISA**	**IFA**	
Bulacan	115/146 (78.8%)	104/146 (71.2%)	119/146 (81.5%)	115/146 (78.8%)	108/146 (74.0%)
Pangasinan	62/69 (89.9%)	54/69 (78.3%)	46/69 (66.7%)	54/69 (78.3%)	46/69 (66.7%)
Tarlac	0/98 (0%)	0/98 (0%)	1/98 (1.0%)	0/98 (0%)	0/98 (0%)
Japan	1/49 (2.0%)	0/49 (0%)	1/49 (2.0%)	0/49 (0%)	0/49 (0%)

### Detection of REBOV neutralizing antibodies in swine

The VSV-pseudotype bearing REBOV-GP efficiently infected Vero E6 cells which are known to be susceptible to REBOV infection. The infectious titer of the VSV-pseudotype reached 3.6 x 10^6^ IU/mL when measured on Vero E6 cells. Infection with the VSV-pseudotype was neutralized with rabbit serum to REBOV-GP (data not shown). Swine sera were analyzed in the NT using the VSV-pseudotype. In the NT, none of the 49 swine sera collected in Japan neutralized the infection with the VSV-pseudotype on Vero E6 cells at serum dilutions of 1 in 100. These were therefore considered to be REBOV NT antibody negative. In the affected farms, 108 (74.0%) of the 146 swine sera in Bulacan and 46 (66.7%) of the 69 swine sera in Pangasinan showed positive reactions in the NT, respectively at a serum dilution of 1 in 100. In total, 154 (71.6%) of the 215 swine sera in the affected farms showed NT antibody positive (Table 1). NT titers of the sera were then obtained for the 34 NT antibody positive sera in the affected farms. NT titers ranged between 100 and 12,800 with average of 790 and median of 400 (data not shown). In Tarlac, none of the 98 swine sera showed any positive reaction (Table 1).

### Detection of REBOV-NP and -GP specific antibodies in swine using IgG-ELISA

**Figure 2:**
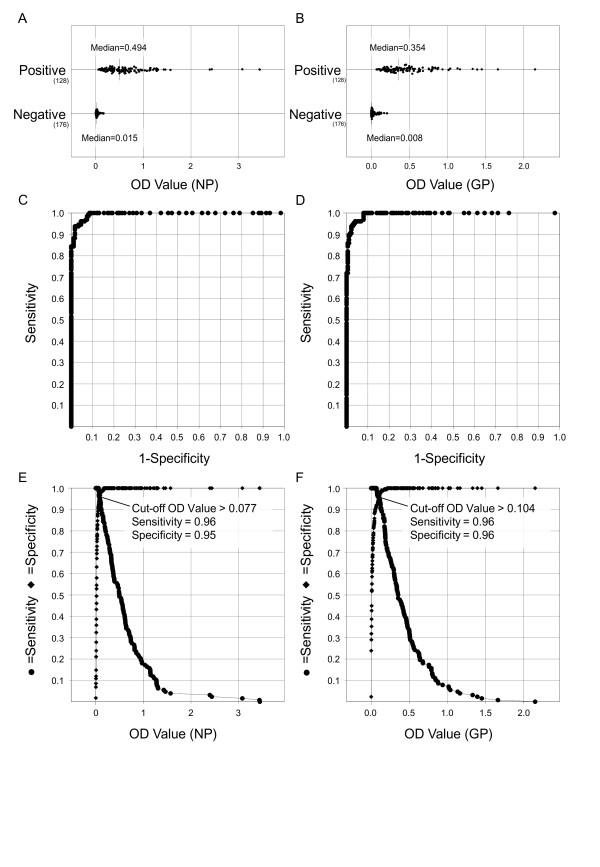
**Distribution of OD values and ROC and TG-ROC analyses of the IgG-ELISA specific for REBOV-NP and -GP.** OD values to the NP (**A**) and GP (**B**) of each serum sample at a dilution of 1 in 100 were plotted for swine group showing either IF and NT antibody positive or negative. ROC and two graph-ROC (TG-ROC) curves were analyzed and drawn using Stat Flex software.

IgG antibodies to the REBOV-NP and -GP were detected by recombinant REBOV protein-based IgG-ELISA. The OD values to the NP and GP of each serum sample at a dilution of 1 in 100 were plotted (Figure 2A and B), and ROC and TG-ROC curves were also drawn (Figure 2C to F). Since the OD values of negative samples were very low, the cut off values defined as the values at intersection points of the sensitivity curve and specificity curve were 0.077 and 0.104 for REBOV-NP and -GP, respectively. At the cut off values, sensitivity and specificity of the assay are over 95% in both IgG-ELISAs (Figure 2E and F). In Bulacan, 115 (78.8%) and 119 (81.5%) of the 146 swine sera showed positive reactions in the NP- and GP-specific IgG-ELISA, respectively. In Pangasinan, 62 (89.9%) and 46 (66.7%) of the 69 swine sera showed positive reactions in the NP- and GP-specific IgG-ELISA, respectively. In total, 177 (82.3%) and 165 (76.7%) of the 215 swine sera collected at the affected farms were REBOV-NP and -GP antibody positive in the IgG-ELISA, respectively (Table 1). Conversely, 1 of the 98 swine sera collected in Tarlac in the Philippines showed a positive reaction in the GP specific IgG-ELISA and 1 of the 49 swine sera collected in Japan showed a positive reaction in the NP- and GP-specific IgG-ELISA (Table 1). However, these results are considered to be false positives because the OD values were close to the cut off level defined by the assay, additionally all the samples collected in Tarlac and Japan tested using the alternative serological assays were negative.

### Agreement between the serological assays detecting REBOV antibodies

We next examined the agreement between the serological assays used in the present study. The data obtained in each assay were binarized to either positive or negative result. These binarized data were then statistically analyzed by pair-wise comparisons in nonparametric one-way ANOVA. This analysis showed that there were no significant differences between the serological assays (p > 0.05, data not shown). The result supports the contention that all of the five serological assays used in the present study posses similar discriminatory capacity. Thus we are confident that many REBOV antibody positive samples showed a positive reaction in all of the five assays.

## Discussion

ZEBOV and Marburg virus were identified in African fruit bat species [19,20]. More recently, Marburg virus was successfully isolated from a fruit bat, *Rousettus aegyptiacus*[21], suggesting that fruit bats are reservoir animals of filoviruses. In the Philippines, we have recently demonstrated that an Asian fruit bat, *Rousettus amplexicaudatus,* has antibodies to REBOV [22]. Since the REBOV genome has not yet been detected in the bat, conclusive evidence that the bat species is a reservoir, or one of the reservoir animals, of REBOV is not available. Nevertheless, it is possible that REBOV was transmitted to swine from these bats since these bats inhabit many areas of the country, including the regions around the affected facilities both in Pangasinan and Bulacan.

In this study, we have aimed to clarify how REBOV infection was spread among swine during the REBOV epizootics. We have tested 215 swine sera collected from the REBOV affected farms using IFA and IgG-ELISA specific for REBOV-NP and -GP, and NT. NT is the gold standard of serological assay in many virus infections. However, since REBOV needs to be cultured in high-containment laboratories, we performed an alternative NT using the VSV-pseudotype bearing REBOV-GP. This avoided the use of infectious REBOV, enabling the work to be carried out at low containment. Previously it has been shown that VSV-pseudotype bearing ebolavirus GP mimicks ebolavirus infection [23], Approxymately 70% of the swine sera from REBOV affected farms were REBOV antibody positive. This indicated that swine are susceptible to REBOV infection. Unfortunately, we could not analyze the IgM antibody responses in swine, since after the sera were heat inactivated the gamma globulin fractions were precipitated with ammonium sulfate, and reconstituted in PBS prior to be testing. An indication of the IgM responses to REBOV would have provided evidence of a recent infection. Thus, it is still unclear if REBOV infection was spread during epizootics or whether a population of the animals in the farms was infected with REBOV prior to the epizootics. The swine not associated with the epizootics, in Tarlac, are considered to be free from REBOV infection. These samples were collected in 2010, over 2 years after the epizootic, and from animals born after the epizootic. Moreover, we could not analyze the swine specimens near the affected farms in 2008. Thus, in this study, it is not clear if REBOV infection in 2008 was limited in the affected farms. Further study is necessary to conclude if the swine population in the Philippines is generally free from REBOV infection.

Recently, it has been shown that the experimental infection of swine with REBOV alone resulted in subclinical infection with rapid clearance of the virus [24]. Alternatively, ZEBOV has been shown to replicate to high titers in experimentally infected swine and to cause severe lung pathology resulting in transmission of the virus to naïve animals [25]. Thus, swine has been shown experimentally to be highly susceptible to ZEBOV infection. Furthermore, some amino acid mutations in NP and/or VP24 in ZEBOV resulted in adaptation of the virus to guinea pigs and mice [26,27]. Thus, we cannot rule out the possibility that mutations introduced in the REBOV genome during serial transmission in swine will result in adaptation of the virus to swine in future. In this regard, a regular serological survey of REBOV infection in swine in the Philippines is desirable. The serodiagnosis systems presented in this study might be useful for such a survey.

## Conclusions

The high prevalence of REBOV infection in swine at the affected farms in 2008 suggests that swine are susceptible for REBOV infection. The multiple serological assays used in the study are thought to be useful for future surveillance of REOBV infection in swine in the Philippines.

## Methods

### Swine serum specimens

**Figure 3:**
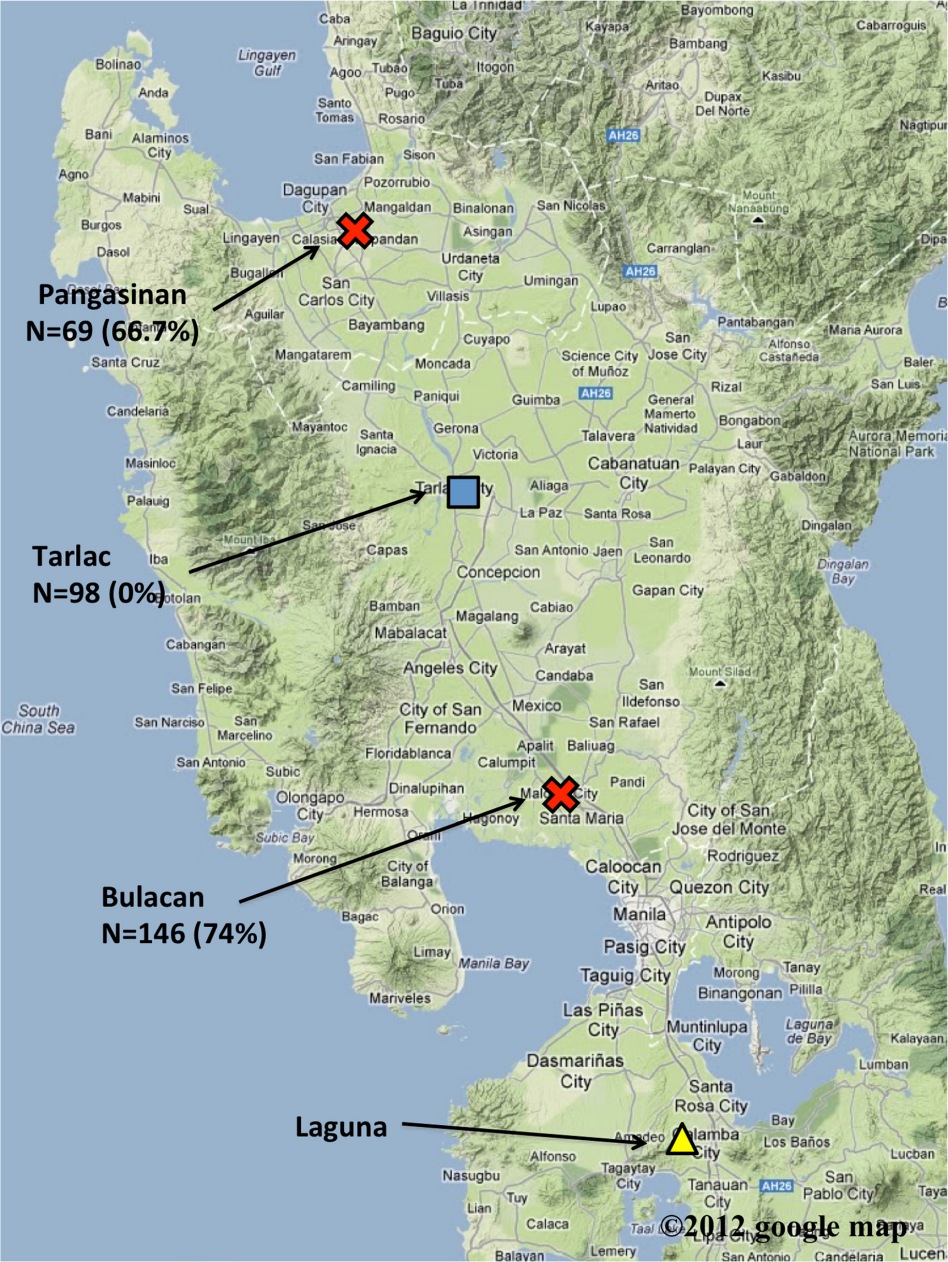
**A topographical map showing locations in the Philippines where swine serum samples were obtained.** Swine serum samples were collected at farms in three regions in the Philippines, Bulacan, Pangasinan and Tarlac. These regions were showed the numbers of specimens and percentage of positivity using NT. The three locations and Laguna, where the Ferlite monkey facility used to be located, are shown on a Google map (©2011 Google Map data ©2011 AND Europa Technologies).

A total of 215 swine sera were collected from two REBOV affected pig farms, located in Pangasinan and Bulacan in 2008 (Figure 3). Of these, 146 sera were collected from swine at the farm in Bulacan, and 69 were collected from those in Pangasinan. Swine samples in the affected farms were collected under quarantine of the Philippines. The sera were kept frozen at the Research Institute for Tropical Medicine (RITM) in the Philippines until use. Ninety-eight swine sera were collected from July to September 2010 from swine aged between 2 and 20 months (median of 4.5 months) in Tarlac in the Philippines, where no swine epizootic has been documented. The swine specimens at Tarlac were collected and used under approval of IRB (No. 2009-018) of RITM, and informed consent was obtained from the farm owners. Forty-nine swine sera collected at slaughterhouse in 2006-7 in Japan, kindly supplied by Dr. T-C Li at the National Institute of Infectious Diseases, were used as REBOV antibody negative control sera. Swine sera were inactivated at 56°C for 30 minutes, and then the gamma globulin fractions were precipitated with ammonium sulfate and reconstituted in phosphate buffered saline (PBS).

### Immunofluorescence assay (IFA) specific to REBOV-NP and -GP using HeLa cells expressing the recombinant protein

The entire cDNA of REBOV-GP ORF was amplified from the swine lymph node specimen used for amplification of the cDNA of NP by RT-PCR using the primers REBOV-GP/F (5’-CGA AGC TTC GAA CAT GGG GTC AGG ATA TCA ACT-3’) and REBOV-GP/R (5’-CGA AGC TTC AAC ACA AAA TCT TAC ATA TAC AAA G-3’) (the *Hind* III site is underlined). The complete nucleotide sequence of the amplicon was determined to be identical to that of Reston 08A (GenBank accession number FJ621583), indicating that the specimen was collected from the same farm as that of sample group A from which Reston 08A was isolated [17]. The *Hind* III fragment of the amplicon was subsequently cloned into pKS336 [28], and the pKS336 plasmid with REBOV-GP, pKS336-pREBOV-GP, was used for transfection. HeLa cells were transfected with pKS336-pREBOV-GP using a FuGENE HD (Roche Diagnostics, Mannheim, Germany) according to the manufacturer’s instructions. The cells transfected with the plasmids were selected with 3 μg/mL of blasticidin S-hydrochloride (Invitrogen, CA, USA) in Dulbecco’s modified Eagle medium (DMEM) supplemented with 5% fetal calf serum (FCS). The selected cells were analyzed for the expression of REBOV-GP in IFA using anti-REBOV-GP rabbit serum. The cells expressing REBOV-GP were spotted on multiwell slide glasses, fixed in acetone, and used as antigens for IFA specific to REBOV-GP. The IFA specific to REBOV-NP was performed as described previously [18], with slight modifications. Mock-transfected HeLa cells were used as negative control antigens in the IFA. The antigen slides were incubated with serially diluted swine sera under humidified conditions at 37°C for 1 hour. The antigen slides were washed in PBS, then reacted with rabbit anti-pig IgG conjugated with fluorescein isothiocianate (FITC) (Bethyl, TX, USA) at a dilution of 1 in 100 at 37°C for 1 hour. The slides were washed in PBS, covered with cover glasses, and examined for staining patterns under a fluorescence microscope fitted with appropriate barrier and excitation filters for FITC visualization. The antibody titer in the IFA was determined as the reciprocal of the highest dilution showing positive staining.

### Generation of vesicular stomatitis virus (VSV)-pseudotype bearing REBOV-GP

Generation of VSV-pseudotype bearing REBOV-GP was performed as described previously [29-31]. Briefly, 293 T cells were transfected with pKS336-pREBOV-GP and cultured for 24 hours, and then the cells were infected with VSVΔG* (kindly supplied by Prof. M.A. Whitt, University of Tennessee) [30,31] and cultured for 24 hours. The culture supernatants were then collected, filtered through a 0.22 μm-pore-size filter, and stored at -80°C until use. The infectious titer (infectious unit, IU) of VSV-pseudotype on Vero E6 cells was determined by counting the number of Green Fluorescent Protein (GFP)-expressing cells.

### Neutralization test (NT)

The sera were diluted twofold from 1 in 100 with DMEM containing 5% FCS and 1,000 IU of VSV-pseudotype bearing REBOV-GP. The mixture was incubated for 1 hour at 37°C, then inoculated onto Vero E6 cells seeded on 96-well plates. The numbers of VSV-pseudotype infected cells were determined by counting of the number of GFP-positive cells according to the methods described previously [23,29,30]. NT titers of the tested sera were defined as the reciprocals of the highest dilutions at which more than 50% inhibition of infectivity was observed. Serum samples were considered to be NT antibody negative when less than 50% inhibition of infectivity was observed at a dilution of 1 in 100.

### Recombinant baculoviruses expressing recombinant REBOV proteins

cDNA of REBOV nucleoprotein (NP) open reading frame (ORF) from a swine lymph node specimen collected at the farm in Bulacan was amplified by reverse transcription polymerase chain reaction (RT-PCR), and the nucleotide sequence of the amplicon was determined to be identical to that of Reston 08A. The cDNA was subcloned into pGEM-Teasy (Promega, WI, USA) and used for the following PCR template. PCR was performed using the plasmid clone to add *Bam*HI linker with primers REBOV-NP/F (5’-GGG CTA GCG GAT CCA AGT CGA TAT GGA TCG TGG GAC C-3’) and REBOV-NP/R (5’-TTG CGG CCG CGG ATC CCT GAT GGT GCT GCA AGA TTG-3’) (the restriction site is underlined). A *Bam*HI-digested fragment of the plasmid was then subcloned into pAcYM1-C-His plasmid, a derivative of pAcYM1 plasmid [32] carrying eight histidine coding sequences just downstream of the *Bam*HI site, to construct pAcYM1-His-pREBOV-NP. A recombinant baculovirus, Ac-His-pREBOV-NP, was generated using a previously described method [33]. The recombinant baculovirus, which expresses the ectodomain of REBOV-glycoprotein (GP) with a histidine-tag at its carboxyl terminus [22], was used to prepare recombinant REBOV-GP for IgG-ELISA. A baculovirus (Ac-ΔP) that lacks the polyhedorin gene was used to prepare a negative-control antigen in insect cells.

### Expression and purification of recombinant NP and GP of REBOV in baculovirus

Tn5 insect cells were infected with Ac-His-pREBOV-NP and Ac-His-REBOV-GP and then incubated at 26°C for 72 hours and 48 hours, respectively. The cells were washed in PBS, lyzed in PBS containing 1% NP40 and 8 M urea, and then clarified by centrifugation at 8,000 rpm for 10 min. The supernatant fraction was collected, and recombinant REBOV-NP and REBOV-GP were purified using a Ni2 + -resin purification system (QIAGEN, Düsseldorf, Germany) according to the manufacturer’s instructions. The expression and purification of REBOV-NP and REBOV-GP were confirmed in 10% sodium dodecyl sulfate polyacrylamide gel electrophoresis (SDS-PAGE) and Coomassie Brilliant Blue R-250 staining Tn5 cells infected with Ac-ΔP were processed similarly and used as negative control antigen in the ELISAs. The purified antigens and negative control antigen were kept at -80°C until use.

### IgG-ELISA

Swine sera were analyzed for the presence of antibodies to REBOV-NP and REBOV-GP in IgG-ELISA, essentially as described previously [22,34,35]. Briefly, half of the wells of the ELISA plates (Falcon, NJ, USA) were coated with a predetermined optimal quantity (approximately 100 ng/well) of each purified recombinant protein, and the rest of the wells were coated with the negative control antigens. After washing the plates three times in PBS containing 0.05% Tween-20 (T-PBS; SIGMA-ALDRICH, MO, USA), each well was incubated with 200 μL of T-PBS containing 5% skim milk (MT-PBS; Yukijirushi, Hokkaido, Japan) for 1 hour at 37°C. After washing the plates three times in T-PBS, the antigen-coated and negative-control-antigen–coated wells were inoculated with the test samples (100 μL/well) in MT-PBS at a dilution of 1 in 100, incubated for 1 hour at 37°C, and washed in T-PBS. Then, each well was incubated with a Protein A/G conjugated with horseradish peroxidase at a dilution of 1 in 1,500 (PIERCE, IL, USA) in MT-PBS for 1 hour at 37°C. The plates were washed three times, and 100 μL of ABTS solution (Roche Diagnostics, Mannheim, Germany) was added to each well. The plates were incubated for 30 min, and the optical density (OD) was measured at 405 nm with a reference at 490 nm. The adjusted ODs for each tested sample were calculated by subtracting the ODs of the negative-control-antigen–coated wells from those of the corresponding REBOV-antigen–coated wells.

### Statistical methods

Sensitivity, specificity and predictive values for positive and negative tests were calculated by standard methods. Sensitivity and specificity were defined as the probability that the target assay result was positive when the IFAs specific to REBOV-NP and -GP and NT showed positive and the probability that the target assay result was negative when the IFAs and NT showed negative, respectively. Receiver operating characteristics (ROC) and two graph-ROC (TG-ROC) curves were analyzed using Stat Flex software (Artech Co. Ltd., Osaka, Japan) [36,37]. To examine the agreement between the serological assays used in the present study, we employed Friedman test with Dunn's post-hoc test [38,39] using GraphPad Prism using pair-wise comparisons in nonparametric one-way ANOVA.

## Competing interests

The authors declare that they have no competing interests.

## Authors’ contributions

YS participated in the study design, the experimental work, the analysis interpretation of the data and drafted the manuscript. CD, MS (Tohoku University), RA, ST, SF, II, TM, IK, FFMJ, SL, DPC, SBA, RGM, ELL, KRCD, MSC, RO, MS and HO participated in the study design, the experimental work, and helped draft the manuscript. TY participated in the statistical analysis of the data. SM conceived and designed the study and participated in the analysis and interpretation of the data and writing of the manuscript. All authors read and approved the final manuscript.

## Authors’ information

YS is a graduate student at Department of Virology, Tohoku University Graduate School of Medicine. CD, FFMJ, SL, and RO are staff at Research Institute for Tropical Diseases. MS and HO are staff at Department of Virology, Tohoku University Graduate School of Medicine. RA, DPC, SBA, RGM, ELL, KRCD, and MSC are staff at Bureau of Animal Industry. ST, SF, TY, II, TM, IK, MS and SM are staff at National Institute of Infectious Disease.

## References

[B1] Feldmann H Klenk HD Sanchez A Molecular biology and evolution of filoviruses Arch Virol Suppl 1993 7 81 100 10.1007/978-3-7091-9300-6_8 8219816

[B2] Ebola haemorrhagic fever in Zaire, 1976 Bull World Health Organ 1978 56 2 271 293 307456 PMC2395567

[B3] Khan AS Tshioko FK Heymann DL Le Guenno B Nabeth P Kerstiens B Fleerackers Y Kilmarx PH Rodier GR Nkuku O The reemergence of Ebola hemorrhagic fever, Democratic Republic of the Congo, 1995. Commission de Lutte contre les Epidemies a Kikwit J Infect Dis 1999 179 Suppl 1 S76 86 9988168 10.1086/514306

[B4] Sadek RF Khan AS Stevens G Peters CJ Ksiazek TG Ebola hemorrhagic fever, Democratic Republic of the Congo, 1995: determinants of survival J Infect Dis 1999 179 Suppl 1 S24 27 9988161 10.1086/514311

[B5] Outbreak of Ebola haemorrhagic fever Uganda, August 2000-January 2001 Wkly Epidemiol Rec 2001 76 6 41 46 11233580

[B6] Towner JS Sealy TK Khristova ML Albarino CG Conlan S Reeder SA Quan PL Lipkin WI Downing R Tappero JW Newly discovered ebola virus associated with hemorrhagic fever outbreak in Uganda PLoS Pathog 2008 4 11 e1000212 10.1371/journal.ppat.1000212 19023410 PMC2581435

[B7] Miranda ME White ME Dayrit MM Hayes CG Ksiazek TG Burans JP Seroepidemiological study of filovirus related to Ebola in the Philippines Lancet 1991 337 8738 425 426 1671441 10.1016/0140-6736(91)91199-5

[B8] Geisbert TW Rhoderick JB Jahrling PB Rapid identification of Ebola virus and related filoviruses in fluid specimens using indirect immunoelectron microscopy J Clin Pathol 1991 44 6 521 522 10.1136/jcp.44.6.521 2066435 PMC496840

[B9] Geisbert TW Jahrling PB Use of immunoelectron microscopy to show Ebola virus during the 1989 United States epizootic J Clin Pathol 1990 43 10 813 816 10.1136/jcp.43.10.813 2229429 PMC502829

[B10] Geisbert TW Jahrling PB Hanes MA Zack PM Association of Ebola-related Reston virus particles and antigen with tissue lesions of monkeys imported to the United States J Comp Pathol 1992 106 2 137 152 10.1016/0021-9975(92)90043-T 1597531

[B11] Hayes CG Burans JP Ksiazek TG Del Rosario RA Miranda ME Manaloto CR Barrientos AB Robles CG Dayrit MM Peters CJ Outbreak of fatal illness among captive macaques in the Philippines caused by an Ebola-related filovirus Am J Trop Med Hyg 1992 46 6 664 671 1621890 10.4269/ajtmh.1992.46.664

[B12] Update: evidence of filovirus infection in an animal caretaker in a research/service facility MMWR Morb Mortal Wkly Rep 1990 39 17 296 297 2109176

[B13] Ikegami T Miranda ME Calaor AB Manalo DL Miranda NJ Niikura M Saijo M Une Y Nomura Y Kurane I Histopathology of natural Ebola virus subtype Reston infection in cynomolgus macaques during the Philippine outbreak in 1996 Exp Anim 2002 51 5 447 455 10.1538/expanim.51.447 12451705

[B14] Rollin PE Williams RJ Bressler DS Pearson S Cottingham M Pucak G Sanchez A Trappier SG Peters RL Greer PW Ebola (subtype Reston) virus among quarantined nonhuman primates recently imported from the Philippines to the United States J Infect Dis 1999 179 Suppl 1 S108 114 9988173 10.1086/514303

[B15] Jahrling PB Geisbert TW Jaax NK Hanes MA Ksiazek TG Peters CJ Experimental infection of cynomolgus macaques with Ebola-Reston filoviruses from the 1989-1990 U.S. epizootic Arch Virol Suppl 1996 11 115 134 8800793 10.1007/978-3-7091-7482-1_11

[B16] Miranda ME Miranda NL Reston ebolavirus in humans and animals in the Philippines: a review J Infect Dis 2011 204 Suppl 3 S757 760 10.1093/infdis/jir296 21987747

[B17] Barrette RW Metwally SA Rowland JM Xu L Zaki SR Nichol ST Rollin PE Towner JS Shieh WJ Batten B Discovery of swine as a host for the Reston ebolavirus Science 2009 325 5937 204 206 10.1126/science.1172705 19590002

[B18] Ikegami T Saijo M Niikura M Miranda ME Calaor AB Hernandez M Manalo DL Kurane I Yoshikawa Y Morikawa S Development of an immunofluorescence method for the detection of antibodies to Ebola virus subtype Reston by the use of recombinant nucleoprotein-expressing HeLa cells Microbiol Immunol 2002 46 9 633 638 12437031 10.1111/j.1348-0421.2002.tb02745.x

[B19] Leroy EM Kumulungui B Pourrut X Rouquet P Hassanin A Yaba P Delicat A Paweska JT Gonzalez JP Swanepoel R Fruit bats as reservoirs of Ebola virus Nature 2005 438 7068 575 576 10.1038/438575a 16319873

[B20] Towner JS Pourrut X Albarino CG Nkogue CN Bird BH Grard G Ksiazek TG Gonzalez JP Nichol ST Leroy EM Marburg virus infection detected in a common African bat PLoS One 2007 2 1 e764 17712412 10.1371/journal.pone.0000764 PMC1942080

[B21] Towner JS Amman BR Sealy TK Carroll SA Comer JA Kemp A Swanepoel R Paddock CD Balinandi S Khristova ML Isolation of genetically diverse Marburg viruses from Egyptian fruit bats PLoS Pathog 2009 5 7 e1000536 10.1371/journal.ppat.1000536 19649327 PMC2713404

[B22] Taniguchi S Watanabe S Masangkay JS Omatsu T Ikegami T Alviola P Ueda N Iha K Fujii H Ishii Y Reston Ebolavirus antibodies in bats, the Philippines Emerg Infect Dis 2011 17 8 1559 1560 21801651 10.3201/eid1708.101693 PMC3381561

[B23] Ito H Watanabe S Takada A Kawaoka Y Ebola virus glycoprotein: proteolytic processing, acylation, cell tropism, and detection of neutralizing antibodies J Virol 2001 75 3 1576 1580 10.1128/JVI.75.3.1576-1580.2001 11152533 PMC114066

[B24] Marsh GA Haining J Robinson R Foord A Yamada M Barr JA Payne J White J Yu M Bingham J Ebola Reston virus infection of pigs: clinical significance and transmission potential J Infect Dis 2011 204 Suppl 3 S804 809 10.1093/infdis/jir300 21987755

[B25] Kobinger GP Leung A Neufeld J Richardson JS Falzarano D Smith G Tierney K Patel A Weingartl HM Replication, Pathogenicity, Shedding, and Transmission of Zaire ebolavirus in Pigs J Infect Dis 2011 204 2 200 208 10.1093/infdis/jir077 21571728

[B26] Volchkov VE Chepurnov AA Volchkova VA Ternovoj VA Klenk HD Molecular characterization of guinea pig-adapted variants of Ebola virus Virology 2000 277 1 147 155 10.1006/viro.2000.0572 11062045

[B27] Ebihara H Takada A Kobasa D Jones S Neumann G Theriault S Bray M Feldmann H Kawaoka Y Molecular determinants of Ebola virus virulence in mice PLoS Pathog 2006 2 7 e73 10.1371/journal.ppat.0020073 16848640 PMC1513261

[B28] Saijo M Qing T Niikura M Maeda A Ikegami T Sakai K Prehaud C Kurane I Morikawa S Immunofluorescence technique using HeLa cells expressing recombinant nucleoprotein for detection of immunoglobulin G antibodies to Crimean-Congo hemorrhagic fever virus J Clin Microbiol 2002 40 2 372 375 10.1128/JCM.40.2.372-375.2002 11825944 PMC153404

[B29] Fukushi S Mizutani T Saijo M Kurane I Taguchi F Tashiro M Morikawa S Evaluation of a novel vesicular stomatitis virus pseudotype-based assay for detection of neutralizing antibody responses to SARS-CoV J Med Virol 2006 78 12 1509 1512 10.1002/jmv.20732 17063504 PMC7166816

[B30] Fukushi S Mizutani T Saijo M Matsuyama S Taguchi F Kurane I Morikawa S Pseudotyped vesicular stomatitis virus for functional analysis of SARS coronavirus spike protein Adv Exp Med Biol 2006 581 293 296 10.1007/978-0-387-33012-9_50 17037546 PMC7122926

[B31] Takada A Robison C Goto H Sanchez A Murti KG Whitt MA Kawaoka Y A system for functional analysis of Ebola virus glycoprotein Proc Natl Acad Sci USA 1997 94 26 14764 14769 10.1073/pnas.94.26.14764 9405687 PMC25111

[B32] Matsuura Y Possee RD Overton HA Bishop DH Baculovirus expression vectors: the requirements for high level expression of proteins, including glycoproteins J Gen Virol 1987 68 Pt 5 1233 1250 3553425 10.1099/0022-1317-68-5-1233

[B33] Kitts PA Ayres MD Possee RD Linearization of baculovirus DNA enhances the recovery of recombinant virus expression vectors Nucleic Acids Res 1990 18 19 5667 5672 10.1093/nar/18.19.5667 2216760 PMC332298

[B34] Saijo M Niikura M Ikegami T Kurane I Kurata T Morikawa S Laboratory diagnostic systems for Ebola and Marburg hemorrhagic fevers developed with recombinant proteins Clin Vaccine Immunol 2006 13 4 444 451 10.1128/CVI.13.4.444-451.2006 16603611 PMC1459631

[B35] Ikegami T Saijo M Niikura M Miranda ME Calaor AB Hernandez M Manalo DL Kurane I Yoshikawa Y Morikawa S Immunoglobulin G enzyme-linked immunosorbent assay using truncated nucleoproteins of Reston Ebola virus Epidemiol Infect 2003 130 3 533 539 12825739 PMC2869991

[B36] Greiner M Sohr D Gobel P A modified ROC analysis for the selection of cut-off values and the definition of intermediate results of serodiagnostic tests J Immunol Methods 1995 185 1 123 132 10.1016/0022-1759(95)00121-P 7665894

[B37] Zweig MH Campbell G Receiver-operating characteristic (ROC) plots: a fundamental evaluation tool in clinical medicine Clin Chem 1993 39 4 561 577 8472349

[B38] Demsar J Statistical Comparisons of Classifiers over Multiple Data Sets Journal of Machine Learning Research 2006 7 1 30

[B39] Dunn OJ Multiple Comparisons Among Means J Am Stat Assoc 1961 56 293 52 64 10.1080/01621459.1961.10482090

